# Clinical workload in UK primary care: a retrospective analysis of 100 million consultations in England, 2007–14

**DOI:** 10.1016/S0140-6736(16)00620-6

**Published:** 2016-06-04

**Authors:** F D Richard Hobbs, Clare Bankhead, Toqir Mukhtar, Sarah Stevens, Rafael Perera-Salazar, Tim Holt, Chris Salisbury, National Institute for Health Research School for Primary Care Research

**Affiliations:** a Nuffield Department of Primary Care Health Sciences, Radcliffe Primary Care Building, Radcliffe Observatory Quarter, Oxford, UK; b Centre for Academic Primary Care, School of Social and Community Medicine, Canynge Hall, Bristol, UK

## Abstract

**Background:**

Primary care is the main source of health care in many health systems, including the UK National Health Service (NHS), but few objective data exist for the volume and nature of primary care activity. With rising concerns that NHS primary care workload has increased substantially, we aimed to assess the direct clinical workload of general practitioners (GPs) and practice nurses in primary care in the UK.

**Methods:**

We did a retrospective analysis of GP and nurse consultations of non-temporary patients registered at 398 English general practices between April, 2007, and March, 2014. We used data from electronic health records routinely entered in the Clinical Practice Research Datalink, and linked CPRD data to national datasets. Trends in age-standardised and sex-standardised consultation rates were modelled with joinpoint regression analysis.

**Findings:**

The dataset comprised 101 818 352 consultations and 20 626 297 person-years of observation. The crude annual consultation rate per person increased by 10·51%, from 4·67 in 2007–08, to 5·16 in 2013–14. Consultation rates were highest in infants (age 0–4 years) and elderly people (≥85 years), and were higher for female patients than for male patients of all ages. The greatest increases in age-standardised and sex-standardised rates were in GPs, with a rise of 12·36% per 10 000 person-years, compared with 0·9% for practice nurses. GP telephone consultation rates doubled, compared with a 5·20% rise in surgery consultations, which accounted for 90% of all consultations. The mean duration of GP surgery consultations increased by 6·7%, from 8·65 min (95% CI 8·64–8·65) to 9·22 min (9·22–9·23), and overall workload increased by 16%.

**Interpretation:**

Our findings show a substantial increase in practice consultation rates, average consultation duration, and total patient-facing clinical workload in English general practice. These results suggest that English primary care as currently delivered could be reaching saturation point. Notably, our data only explore direct clinical workload and not indirect activities and professional duties, which have probably also increased. This and additional research questions, including the outcomes of workload changes on other sectors of health care, need urgent answers for primary care provision internationally.

**Funding:**

Department of Health Policy Research Programme.

## Introduction

Primary care is central to the provision of health care in many developed health systems, including the National Health Service (NHS), providing most first-contact health care; disease diagnosis, monitoring, and management; and disease prevention, through screening and health promotion. In many countries, primary care is also the gatekeeper to secondary care in hospital, and can provide sickness certification access to benefits. However, despite this high volume of health-care activity internationally (about 90% of all NHS contacts occur in primary care), remarkably few data are available for the volume and nature of primary care activity from any country.

Most estimates of primary care workload are from the UK, but are either very old,^1^ only provide crude consultation rates (up to 2009),^2^ or are based on survey recall.^3^ In the past few years, concerns have increased, especially in the UK, that primary care is overwhelmed by unsustainable workload increases, with pressures on emergency departments reported as due to reduced access to general practice. There are also rising difficulties in recruitment to general practice vacancies and training posts. The 2015 general practitioner (GP) worklife satisfaction survey showed the lowest overall job satisfaction since the surveys started in 2001.^4^ We therefore did this study to obtain accurate data for the volume and nature of primary care workload.

## Methods

### Study design and population

We did this retrospective analysis of GP and nurse consultations of non-temporary patients registered at 398 English general practices between April, 2007, and March, 2014. Analysis was not restricted to patients who were continuously registered throughout the study period. We used data from the Clinical Practice Research Datalink (CPRD)—a large database of patient-level anonymised primary care electronic health records. Data are available for 674 practices covering 6·9% of the UK population,^5^ including 11·3 million patients (4·4 million alive) shown to be broadly representative with regard to age, sex,^5^ and ethnic origin.^6^ The CPRD includes only practices meeting quality checks on completeness and reliability of the data.^7^ We excluded so-called unacceptable data (ie, data not meeting quality criteria set by CPRD) in the database, since 90% of these data are linked to temporary patients.^7^

**Research in context**

**Evidence before this study**

Few data exist for workload in primary care internationally. We searched PubMed on Sept 4, 2015, and again on Feb 15, 2016, with the search terms “workload”, “primary care”, and “general practice”. We also checked citations. All relevant English-language publications were considered with no methodological or quality restrictions imposed. Most evidence is based on surveys of practitioners or patients (mainly in the UK, Australia, and Europe), with a few observational studies (usually of workload associated with certain disease presentations), or very few trials of differing ways of primary care delivery and effect on trial-measured workload (eg, telephone triaging in the ESTEEM trial; Campbell JL et al, 2014). Objective data for clinical workload in primary care are scarce and, in the UK, were last published in 2008, and only for overall consultation numbers. By contrast, increased published data exist for physician perceptions of rapidly rising demand and unsustainable workload. Data matching perceptions of workload against evidence for activity are therefore needed.

**Added value of this study**

This study reports the most recent objective data for primary care consultation numbers and consultation rates (crude and population adjusted), and is the first to analyse duration of consultations. Our findings show a substantial increase in practice consultation rates, average consultation duration, and total patient-facing clinical workload in English general practice over 7 years between 2007 and 2015. These findings are based on the largest analysis of consultations, more than 20 million person-years of observation, from one of the most validated databases of routinely collected electronic clinical records in the world (the Clinical Practice Research Datalink).

**Implications of all the available evidence**

Increasing subjective evidence of rising workload in international primary care, especially in the UK, is borne out in this objective analysis. Direct clinical workload in primary care has increased consistently in the UK, over and above the growth in the population and the growth in general practitioner (GP) and nurse numbers, and is likely to continue growing. Perceptions of unsustainability of this workload from GP surveys in the UK also seem to have an objective evidence base, since the mean duration of face-to-face consultations is approaching the maximum duration available for most booked consultations in the UK—ie, that the system might be approaching saturation. Evidence also showed that one response to coping with this increased workload—use of telephone triage—is accelerating rapidly (doubled in the 7 years), despite findings showing that this strategy does not reduce overall primary care workload, only appeals to a subset of the population, and might reduce the proportion of primary care devoted to preventive activities. These data confirm a mechanism to provide ongoing workload statistics for health planning. More research is needed into the outcomes of workload changes on other sectors of health care, such as admissions, on prescribing or investigation trends, or linked to disease states or comorbidities. The present data also provide a mechanism to generate workforce numbers that might be predicted are needed to cover demand from patients, based on specific population demographics. All these additional research questions need urgent answers for primary care provision internationally.

CPRD research is covered by a broad National Research Ethics Service ethics approval system. This study was approved by the CPRD Independent Scientific Advisory Committee.

### Procedures

We linked CPRD data to national datasets of two types: datasets routinely available with CPRD, such as deprivation data based on the English Index of Multiple Deprivation, and bespoke linkages at practice level to staffing data,^8^ rurality,^9^ patient satisfaction,^10^ and Quality and Outcomes Framework^11^ scores for 2013–14. Because the bespoke linkages could increase the risk of unintentional (deductive) disclosure of specific GP practices and we were obliged to receive aggregate data in quintiles.

CPRD data are clinical entries made routinely in primary care electronic health records by GPs and practice staff (practice nurses, health-care assistants, and administrative staff) coded to type of contact, using a total of 51 codes. We categorised these codes into five entry types: face-to-face surgery consultations, telephone contacts, home visits, administrative, and other. Staff roles, based on login details, are coded using 67 roles, which we categorised into four roles: GP, nurse, other clinicians, and administrative. Here we report on GP and practice nurse direct patient contacts in face-to-face or telephone consultations and home visits. All the recorded activity and timings (start and end time of consultations) represent accurately collected workload data, but might not capture all the time involved with each patient contact.

We calculated person-years of observation for each age and sex strata over the 7 years. Crude rates were calculated for each year of observation and, for comparisons across years, rates were age-standardised and sex-standardised to the 2013 mid-year English population.^12^ We calculated percentage changes from the first year (2007–08) to the last year (2013–14).

CPRD round down contact entries to the nearest whole minute—eg, 8 min 59 s is recorded as 8 min. Consultation durations of less than 1 min are recorded in CPRD data as zero. For analyses, we assumed these durations to be of 30 s. After examination of the distribution of consultation durations, those lasting longer than 60 min were truncated at 60 min. Such entries might be artifacts, for example not closing a record at the end of a clinic, but could still be accurate, for example if an urgent admission had to be arranged. However, this censoring only affected 0·9% of consultations.

Notably, the duration of home visits represents time spent recording what happened on visits, rather than the actual time spent doing the visit, which provides a substantial underestimate of visit duration. We examined duration data for each year and calculated mean duration for each age and sex strata. We calculated changes in duration between the first year (2007–08) and the last year (2013–14) for each type of staff and contact.

### Statistical analysis

Trends in age-standardised and sex-standardised consultation rates were modelled with joinpoint regression,^13^ which identified the estimated location of any significant change in the slope of a trend line.^14^ Each joinpoint represents a year with an estimated change of trend in rates. A maximum of one joinpoint was allowed for each model considered; this was the default value according to the number of observations in each model. The overall significance level for permutation tests was α=0·05. We estimated the annual percentage change for each trend line, with associated confidence intervals. A confidence interval could not be calculated if there were too few datapoints in a line segment.

We calculated a composite measure of total patient facing workload for GPs and nurses by multiplying the age-specific, sex-specific, and year-specific consultation rate for each stratum by the average duration of consultations in minutes for those strata. These calculations were then totalled for each year to provide an estimate of the total number of patient contact hours per 10 000 person-years. This figure was then expressed as number of clinical days per 10 000 person-years by dividing the total time by a notional 6 h (estimated duration of two booked sessions per day).

We did analysis with Stata (version 14), Microsoft Excel 2010, and Joinpoint Regression Programme (version 4.2.0.2).^13^

### Role of the funding source

The funder of the study provided feedback from the international peer review of the protocol, but had no further role in the study design, data collection, data analysis, data interpretation, or writing of the report. TM and SS had full access to all the data in the study.

## Results

Overall, 398 practices were included, with around 360 practices contributing to the first 4 years of observation, declining to 316 practices by 2013–14. The practices tended to be large, with mean list sizes of more than 9600 patients in 2014, from 9000 patients in 2007 (appendix). The dataset comprised 101 818 352 consultations and 20 626 297 person-years of observation (appendix). Contributing practices were located across England, although mostly in southern regions, with 4·65 full-time equivalent GPs on average (excluding registrars), and 154 (40%) of 386 practices were training practices (appendix). Practices were slightly over-representative of those achieving high QOF scores (appendix).

Age-specific consultation rates had a J-shaped distribution, with the highest rates in infants aged 0–4 years, decreasing to the lowest levels in the 15–24-year age group in male patients and the 5–14-year age group in female patients, before rising in each age band, reaching a peak in patients older than 85 years (table 1). Age and sex patterns of consultation rates were consistent across all years (table 1). Consultation rates were slightly higher in boys than girls in the 0–4-year age group, but were significantly higher in women than men at ages 15–44 years, which probably represents consultations regarding contraception, maternal, and prenatal health. In terms of crude consultation numbers, most consultations were in adults aged 25–74 years (62% in 2013–14), mainly due to the numbers in the population in these groups (appendix). After standardisation of age and sex to the 2013 mid-year English population, consultation rates increased by 9·1% over the 7 years, with statistically significant, but constant, annual percentage increases of 1·4% (95% CI 1·1–1·8; figure).

For GPs, overall standardised consultations increased significantly by 12·36% over the 7 years (figure, appendix). Surgery consultations, which accounted for most GP consultations, increased by 5·20% (appendix), with significant increases for the first 5 years (1·3% per year, 95% CI 1·3–1·4) followed by a non-significant decrease of 1·6% (figure). The biggest change was in GP telephone consultations rates, which increased by 99·6% over 7 years (appendix), with a constant significant change of 11·1% per year (95% CI 7·8–14·5 figure). Conversely, GP visit rates decreased steadily by a small but significant −1·8% (95% CI −2·5 to −1·1) per year, but represent only 2% of consultations.

For practice nurses, overall age-adjusted and sex-adjusted consultation rates in the first 2 years showed a non-significant increase of 2·1% per year (95% CI −1·1 to 5·3) then a non-significant decrease of −0·8% per year (−1·8 to 0·3; figure). Face-to-face nurse consultations remained stable, whereas telephone rates showed a slight, non-significant decrease of 0·6% per year (95% CI −2·5 to 1·3). Nurse home-visit rates decreased by −6·5% per year (95% CI −8·8 to −4·1) for the first 5 years.

The consultation rate per patient per year in English primary care rose by 10·51% from 2007–08 to 2013–14 (table 2). Most of the increase was in GP consultations, which increased by 13·67% over the 7 years (table 2).

The mean duration of overall consultations with GPs or nurses was 8·86 min by 2013–14, representing a 4·94% (95% CI 4·82–5·06) increase (or 25 s) over the 7 years (table 3). Larger increases were shown at the extremes of the age range, with increases of 9·3% in children aged 0–4 years and 10·1% in adults aged 85 years and older (table 3). Patients aged 45–64 years consistently had the longest consultations (table 3). Most increases in consultation duration happened in the first 4 years, with a 1·8% (95% CI −0·4 to 4·1) non-significant annual percentage rise until 2010–11, when the rate of increase plateaued. The increase in mean duration of consultations overall was moderated by the increasing proportion of GP telephone consultations, which had a mean duration of 5·4 min (95% CI 5·3–5·4) in 2013–14. The mean duration of GP face-to-face surgery consultations increased significantly by 6·7%, from 8·65 min (95% CI 8·64–8·65) in 2007–08, to 9·22 min (9·22–9·23) in 2013–14 (appendix).

Nurse face-to-face consultations were of a similar duration to those of GPs at 9·72 min (95% CI 9·71–9·73). This time slowly, but significantly, increased at an annual percentage change of 1·08% (95% CI 0·4–1·7) each year. The duration of nurse telephone consultations steadily and significantly increased by 2·8% (95% CI 2·2–3·4) each year, reaching a mean duration of 5·69 min (95% CI 5·66–5·72) in 2013–14.

The number of clinical days needed for consulting per 10 000 person-years increased by 16·0% over the 7 years (table 4). We recorded a U-shaped association with age, with the lowest increases in 25–74 year olds and the largest increases in the patients older than 85 years most elderly people (table 4). There were increases of 3·8% per year (95% CI 1·9–5·6) per year in the first 4 years, with a non-significant increase of 1·2% (−0·7 to 3·0) per year thereafter.

Of the 1270 days of direct patient contact time in 2013–14, 913 (95% CI 911·4–914·8; 72%) days represented GP consultations, with an 18·2% increase in days over the 7 years (appendix). Time spent doing GP face-to-face consultations increased by 13·5%, and total time spent doing telephone consultations by GPs more than doubled, from 39 days (95% CI 38·7–29·1) per 10 000 person-years in 2007–08, to 80 days (79·5–80·3) per 10 000 person-years in 2013–14. The number of days of nurse face-to-face consultation time also increased, by 11·4%, although this increase represented a smaller proportion of the overall workload (342 days [95% CI 341·0–343·5; 27% of total direct patient consulting time).

There was little association between consultation rates and deprivation at the practice level, which might have resulted from the aggregation step. We therefore did an exploratory univariate analysis of Index of Multiple Deprivation scores at the patient level, which showed a strong association between deprivation and overall consultation rate (appendix).

## Discussion

These are the first comprehensive data for the direct clinical workload of GPs and practice nurses in primary care, analysing both duration and rates of consultation over 2·5 million patient-years. Number and duration of consultations increased between 2007 and 2014, leading to substantial increases in workload, especially for GPs.

The number of consultations per patient per year in English primary care rose by roughly 10% between 2007–08 and 2013–14. Most of these consultations were face-to-face contacts with GPs in the surgery. The English population has also rapidly increased, by 5·7% from 51·4 million in 2008, to 54·3 million in 2014.^15^ This overall population rise was additionally associated with a disproportionate rise in elderly people and infants younger than 5 years (UK had highest birthrate in Europe in 2012), with both age extremes associated with significantly higher consultation rates, and rate of rise, than any other group. In patient-level univariate analysis, rates of consultation in patients in the most deprived quintile were 3·5% higher than those in patients in the middle quintile and 15% higher than those in the least deprived quintile.

The increasing number of consultations per patient, and the increasing number of patients, has been compounded by a decrease in the number of full-time equivalent GPs per 100 000 patients. On the basis of national data, the number of GPs in England (full-time equivalent, excluding registrars and retainers) rose by 5·5% between 2007 and 2014, from 30 936 to 32 628.^16^ After accounting for population growth, these data represent a 1% decrease in full-time equivalent GPs per 100 000 patients (from 60·9 to 60·6 GPs). Full-time equivalent numbers of practice nurses increased slightly by 3·5%, from 14 554 in 2007, to 15 062 in 2014.

In our study, we recorded slightly lower overall consultation rates and rate of rise in consultations than shown in the only previous study of consultation rates between 1995–96 and 2008–09,^2^ from which data were extrapolated to suggest 340 million primary care consultations per annum in 2013.^17^ These adjusted consultation rates per practitioner show workload changes over and above any extra demand from population growth. We recorded consistent rises in age-standardised and sex-standardised consultation rates over the 7 years, equivalent to a 1·4% annual rise in overall consultations. The biggest increase (12·4%) was in GP consultation rates. These increases comprised small reductions in visit rates, moderate rises in face-to-face consultations, and huge rises in telephone consultations. All the annual rises driving these increases were statistically significant, but with a flattening of the increase in face-to-face consultations in the last year. For practice nurses, the rise in consultations was less substantial, with a 0·9% increase over 7 years and flattening of the rates from 2009. The rise for nurses is based on a small (3%) increase in face-to-face consultations, with reductions in telephone consultations of a tenth and in visit consultations of almost half.

For the first time, we report on the duration of consultations by type. We recorded a small (5%) but consistent increase in consultation time for practitioners during the period of observation. Average face-to-face consultation times with an English GP rose by 7% for GPs and by 6% for nurses. These duration data are important. First, they show multiple effects of rising workload: the 12·4% increase in GP consultation rates linked to a 4% rise in mean consultation duration, shows that GP direct clinical workload has risen by 18·2% in 7 years. The equivalent increases for overall GP and practice nurse consultations were 9·1% for consultation rates and 5% for duration, showing an overall 16% increase in overall measurable direct clinical workload. Second, the data suggest that a common strategy advocated for coping with rising workload—use of telephone triage—has been widely deployed by GPs in England, but has become less efficient, with a doubling of telephone consulting rates over 7 years and small rises in the time needed to do these consultations. The increases in time needed for telephone consultations is important because the evidence for same-day telephone triage shows that it does not reduce overall workload,^16^ due to the time involved in telephone calls (60% of the time seeing the patient face to face) and the proportion (about a third) of calls resulting in a subsequent surgery consultation.^18^

Our findings show that perceptions of rapidly rising workload in English general practice are well founded. The reason why practitioners feel so pressured might be because the overall system seems to be approaching saturation. Most English practices offer patients 10 min appointment slots. These timings are based on an expectation that some consultations will be shorter than others, or that patients will not attend and therefore some longer consultations can be accommodated within the booked clinic length. Because the mean face-to-face consultation time is now approaching 10 min, the GP or nurse will eventually have to consult throughout the booked clinic length, with no break. This situation will be undoubtedly demanding, in view of the various clinical problems being dealt with.

Our study has several limitations. We can only report on time spent recording consultations. Staff types are likely to be reliable being based on individuals' unique login details. For patient visits, the electronic health record is only opened to record what was done during the visit and does not capture time with the patient. The start and end times of most surgery consultations (which accounted for most consultations) are probably accurate being consecutive consultations, but we adjusted for failure to close a record by censoring all consultations at 1 h; less than 1% of consultations exceeded this time and some consults might take more than 1 h. Furthermore, because CPRD round down contact entries to the nearest whole minute, these consultation duration data are likely to be systematically underestimated.

Although the CPRD database is representative of the UK population, we only assessed workload for English practices (a sponsor requirement). The practices included represent around 4·5% of English practices, but were 35% larger on average (2014 mean CPRD list size of 9650 compared with 7171 for all English practices).^8^

The biggest limitation is that data are only available for consultations involving direct patient contact: no data are available for time spent on patient-related clinical activity consequential on consultations, such as arrangement of hospital referrals. We also cannot quantify other professional activities, such as teaching, audit, or continuing professional development. These aspects of GP workload are likely to have also increased substantially since 2007. Because the 2015 GP worklife survey reported that direct patient care accounts for 62% of GP's work time,^4^ our data could under-represent total workload by more than 40%.

In conclusion, English general practice has absorbed consistent major rises in workload relating to direct patient care, with particularly marked increases for GPs in surgery and especially telephone consultations. There are few rapid solutions to workload pressures in primary care. Immediate steps might include increasing the availability of practitioner consulting time by reducing the non-direct clinical workload for a period. NHS plans to expand GP numbers by 5000 will take time and are dependent on an improved appeal of general practice as a career choice for which the major drivers are perceived status and reward for the specialty. The perception of general practice as a stressful, work-pressured, low-status career choice with excessive administration^19^ needs to change. Unfortunately, one main response to workload pressures—more telephone triaging—might help cope with demand, but could undermine some key roles of general practice in disease prevention. One important focus for the NHS could be strategies to reduce patient health-seeking behaviours and increase self-management. If the English population continues to rise overall, as is predicted, and proportions of elderly and young people rise disproportionately, the rises in consultation rates are likely to accelerate.

**This online publication has been corrected. The corrected version first appeared at thelancet.com on June 2, 2016**

## Figures and Tables

**Figure:**
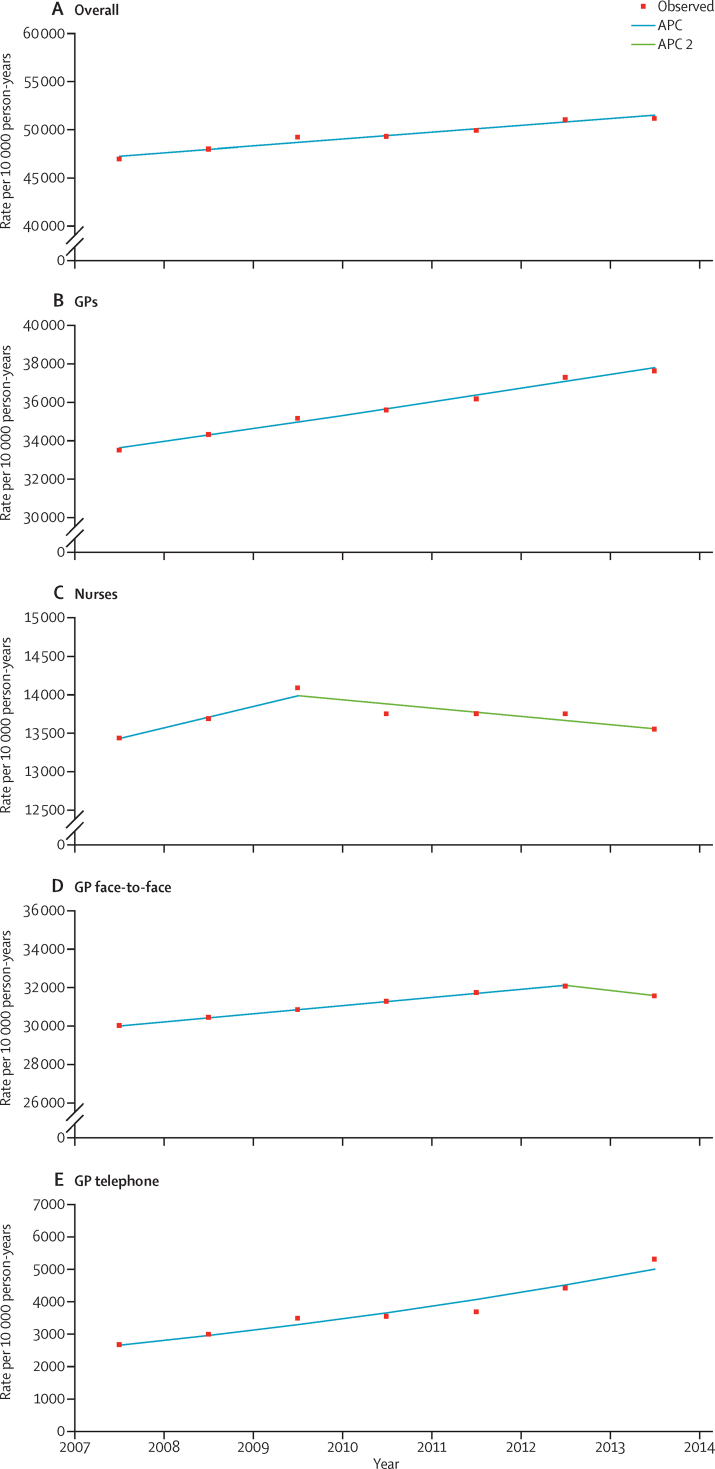
Joinpoint analyses of age-adjusted and sex-adjusted consultation rates Overall; no joinpoints; APC=1·43 (A). In GPs; no joinpoints; APC=1·97 (B). Nurses; one joinpoint; APC 1=2·06, APC 2=–0·76 (C). GP face-to-face consultation; one joinpoint; APC 1=1·34, APC 2=–1·64 (D). GP telephone consultation; no joinpoints; APC=11·11 (E). APC=annual percentage change. GP=general practitioners.

**Table 1:** Crude and adjusted consultation rates (per 10 000 person-years) with a general practitioner or nurse, by age, sex, and year

		**2007–08**		**2008–09**		**2009–10**		**2010–11**		**2011–12**		**2012–13**		**2013–14**	
		Male	Female	Male	Female	Male	Female	Male	Female	Male	Female	Male	Female	Male	Female
Age group (years)															
	0–4	55930·13	52717·93	57206·46	54008·01	59537·55	56609·40	58145·90	54866·96	57181·31	53849·17	59068·59	55841·87	60060·89	56982·03
	5–14	18519·66	20005·54	19329·66	21171·43	19894·15	21684·72	19879·23	21857·67	19541·82	21645·21	20877·81	22953·59	20596·17	22726·34
	15–24	17890·70	42033·23	18702·56	44011·09	19007·89	46654·50	18803·82	45239·32	18982·74	44609·25	19621·55	45443·95	19736·37	45520·48
	25–44	23149·36	50498·99	23586·68	52267·86	24457·50	53642·69	24493·81	54130·93	24973·44	54787·73	25713·89	55550·21	25479·18	55492·36
	45–64	40019·50	56371·87	40605·72	57022·93	41615·89	57873·02	41774·05	58872·00	42557·35	59827·80	43742·84	60727·49	43419·79	60827·87
	65–74	73786·99	80334·79	73958·05	80608·02	75557·58	81322·62	75146·13	81242·72	75916·98	81748·43	76682·35	81964·12	77179·41	82336·15
	75–84	98241·22	100929·99	100154·77	102432·66	102274·30	104474·65	102657·83	105603·97	104739·74	108440·90	107371·59	110780·19	108983·39	112955·90
	≥85	112032·22	108671·79	117879·76	111918·25	120652·79	114818·43	122741·04	119131·83	127525·83	123904·87	131683·87	129122·06	133099·84	131349·12
Total		37553·01	55738·74	38470·85	57038·48	39685·42	58439·99	39791·43	58807·72	40528·77	59498·21	41865·64	60631·60	42055·00	60909·42
Adjusted consultation rate (both sexes combined)		46927·39* (46902·99–46951·80)	..	47962·82 (47938·23–47987·41)	..	49192·30 (49167·48–49217·13)	..	49323·56 (49298·51–49348·60)	..	49884·84 (49859·30–49910·38)	..	51013·95 (50987·82–51040·09)	..	51191·69* (51164·23–51219·15)	..

Data in parentheses are 95% CIs. Crude rates were calculated for each year of observation and, for comparisons across years, rates were age-standardised and sex-standardised to the 2013 mid-year English population.

* An increase of 9·09%.

**Table 2:** Temporal trends in GP and nurse consultations by subtype of face-to-face, telephone, and home visit contacts

		**2007–08**	**2008–09**	**2009–10**	**2010–11**	**2011–12**	**2012–13**	**2013–14**	Percentage change from 2007–08 to 2013–14*
All GP and nurse consultations		4·67	4·78	4·91	4·94	5·01	5·14	5·16	10·51%
All GP consultations		3·35	3·43	3·52	3·57	3·64	3·76	3·80	13·67%
	Face-to-face, surgery	2·99	3·04	3·08	3·13	3·18	3·23	3·18	6·38%
	Telephone	0·27	0·30	0·35	0·36	0·37	0·45	0·54	100·91%
	Home visit	0·09	0·09	0·09	0·09	0·08	0·08	0·08	−6·47%
All nurse consultations		1·32	1·35	1·39	1·37	1·37	1·38	1·36	2·76%
	Face-to-face, surgery	1·21	1·24	1·28	1·26	1·26	1·27	1·27	4·91%
	Telephone	0·08	0·08	0·07	0·08	0·08	0·08	0·07	−9·32%
	Home visit	0·04	0·04	0·03	0·03	0·03	0·03	0·02	−44·11%

Crude rates (per person-year). GP=general practitioner.

* Percentages are based on full data (to greater precision than shown).

**Table 3:** Duration of consultations (min) with all types of general practitioner or nurse

		**2007–08**		**2008–09**		**2009–10**		**2010–11**		**2011–12**		**2012–13**		**2013–14**		Percentage change from 2007–08 to 2013–14*		**Change (s)**	
		Male	Female	Male	Female	Male	Female	Male	Female	Male	Female	Male	Female	Male	Female	Male	Female	Male	Female
Age group (years)																			
	0–4	7·60 (0·010)	7·56 (0·010)	7·86 (0·010)	7·77 (0·010)	7·81 (0·009)	7·71 (0·010)	8·19 (0·010)	8·06 (0·010)	8·28 (0·010)	8·23 (0·010)	8·32 (0·010)	8·23 (0·010)	8·32 (0·011)	8·25 (0·011)	9·45%	9·23%	43·09	41·85
	5–14	7·32 (0·012)	7·39 (0·011)	7·52 (0·012)	7·57 (0·011)	7·59 (0·012)	7·64 (0·011)	7·81 (0·012)	7·88 (0·012)	7·84 (0·012)	7·87 (0·012)	7·75 (0·012)	7·86 (0·012)	7·77 (0·013)	7·86 (0·013)	6·27%	6·44%	27·53	28·54
	15–24	7·90 (0·013)	8·40 (0·009)	8·11 (0·013)	8·59 (0·009)	8·19 (0·013)	8·62 (0·008)	8·46 (0·013)	8·90 (0·009)	8·52 (0·013)	8·96 (0·009)	8·40 (0·013)	8·87 (0·009)	8·36 (0·014)	8·86 (0·010)	5·80%	5·46%	27·51	27·51
	25–44	8·63 (0·008)	8·83 (0·005)	8·88 (0·008)	9·06 (0·005)	8·91 (0·008)	9·09 (0·005)	9·12 (0·008)	9·26 (0·005)	9·15 (0·008)	9·24 (0·005)	8·99 (0·008)	9·09 (0·005)	8·94 (0·009)	9·03 (0·006)	3·58%	2·28%	18·54	12·07
	45–64	8·97 (0·006)	8·94 (0·005)	9·18 (0·006)	9·14 (0·005)	9·19 (0·006)	9·19 (0·005)	9·42 (0·006)	9·38 (0·005)	9·42 (0·007)	9·38 (0·005)	9·33 (0·007)	9·27 (0·006)	9·28 (0·007)	9·28 (0·006)	3·48%	3·77%	18·74	20·21
	65–74	8·46 (0·009)	8·42 (0·008)	8·73 (0·009)	8·65 (0·008)	8·75 (0·008)	8·71 (0·008)	9·03 (0·009)	8·93 (0·008)	9·02 (0·009)	8·90 (0·008)	8·97 (0·009)	8·84 (0·008)	9·01 (0·009)	8·91 (0·008)	6·54%	5·83%	33·19	29·45
	75–84	8·33 (0·010)	8·20 (0·008)	8·57 (0·010)	8·46 (0·008)	8·56 (0·010)	8·52 (0·008)	8·86 (0·010)	8·76 (0·008)	8·87 (0·010)	8·76 (0·009)	8·81 (0·010)	8·70 (0·009)	8·89 (0·011)	8·79 (0·009)	6·74%	7·25%	33·65	35·67
	≥85	7·65 (0·018)	7·15 (0·012)	7·87 (0·017)	7·39 (0·012)	7·94 (0·017)	7·53 (0·012)	8·18 (0·017)	7·65 (0·012)	8·23 (0·017)	7·74 (0·012)	8·23 (0·017)	7·72 (0·012)	8·41 (0·018)	7·88 (0·013)	9·98%	10·14%	45·82	43·52
Total (95% CI; both sexes combined)		8·45 (8·44–8·45)	..	8·67 (8·66–8·67)	..	8·70 (8·70–8·70)	..	8·93 (8·92–8·93)	..	8·94 (8·94–8·95)	..	8·85 (8·85–8·86)	..	8·86 (8·86–8·87)	..	4·94% (4·82–5·06)	..	25·01 (24·41–25·61)	..

Data in parentheses are SEs, unless otherwise specified.

* Percentages are based on full data (to greater precision than shown).

**Table 4:** Total number of days (two × 3 h sessions) per 10 000 person-years of face-to-face, telephone, or visit consultations with a general practitioner or nurse

		**2007–08**		**2008–09**		**2009–10**		**2010–11**		**2011–12**		**2012–13**		**2013–14**		**Percentage from 2007–08 to 2013–14**		**Change (days)**	
		Male	Female	Male	Female	Male	Female	Male	Female	Male	Female	Male	Female	Male	Female	Male	Female	Male	Female
Age group (years)																			
	0–4	1180·6 (4·11)	1106·5 (3·93)	1249·7 (4·23)	1166·3 (4·08)	1291·7 (4·32)	1212·5 (4·17)	1322·5 (4·50)	1228·7 (4·26)	1315·9 (4·55)	1230·6 (4·31)	1364·7 (4·75)	1276·9 (4·53)	1387·6 (5·12)	1306·4 (4·93)	17·5%	18·1%	207·0	199·9
	5–14	376·3 (1·41)	410·6 (1·58)	403·8 (1·50)	445·3 (1·66)	419·3 (1·53)	460·1 (1·73)	431·2 (1·60)	478·2 (1·84)	425·7 (1·63)	473·3 (1·87)	449·5 (1·72)	501·2 (2·00)	444·8 (1·80)	496·5 (2·15)	18·2%	20·9%	68·5	85·9
	15–24	392·6 (1·73)	980·7 (3·09)	421·3 (1·80)	1049·9 (3·18)	432·3 (1·83)	1116·7 (3·25)	441·9 (1·94)	1118·3 (3·42)	449·2 (2·03)	1110·8 (3·51)	458·0 (2·06)	1119·8 (3·55)	458·2 (2·16)	1120·0 (3·80)	16·7%	14·2%	65·6	139·3
	25–44	555·1 (1·54)	1238·7 (2·36)	581·8 (1·62)	1315·6 (2·46)	605·4 (1·68)	1355·0 (2·53)	620·3 (1·75)	1392·2 (2·66)	635·0 (1·85)	1406·9 (2·73)	641·9 (1·94)	1402·4 (2·78)	632·8 (2·03)	1392·2 (2·93)	14·0%	12·4%	77·7	153·5
	45–64	996·9 (2·55)	1400·5 (2·86)	1035·9 (2·57)	1447·5 (2·92)	1062·5 (2·60)	1476·7 (2·99)	1092·9 (2·66)	1534·7 (3·13)	1113·9 (2·82)	1558·2 (3·29)	1133·9 (2·95)	1563·5 (3·29)	1119·3 (3·06)	1568·1 (3·46)	12·3%	12·0%	122·4	167·6
	65–74	1733·1 (6·42)	1878·7 (6·51)	1793·0 (6·49)	1936·0 (6·42)	1836·0 (6·30)	1967·8 (6·33)	1885·4 (6·69)	2015·3 (6·68)	1901·8 (6·95)	2021·0 (6·81)	1909·8 (6·94)	2012·0 (6·77)	1931·4 (7·27)	2037·8 (7·11)	11·4%	8·5%	198·3	159·1
	75–84	2271·9 (10·06)	2298·5 (8·39)	2383·3 (10·57)	2408·0 (8·94)	2432·1 (10·61)	2473·3 (9·20)	2525·7 (11·62)	2571·0 (9·67)	2579·3 (12·32)	2639·6 (10·16)	2628·7 (12·36)	2677·7 (10·36)	2690·0 (12·73)	2758·8 (11·31)	18·4%	20·0%	418·2	460·4
	≥85	2380·4 (19·23)	2159·7 (11·98)	2575·9 (21·03)	2299·0 (12·36)	2659·8 (20·47)	2402·3 (13·05)	2789·0 (21·20)	2531·913·34)	2916·1 (22·07)	2665·2 (14·34)	3008·7 (22·85)	2769·8 (14·80)	3110·4 (24·44)	2875·0 (16·28)	30·7%	33·1%	730·0	715·3
Total (95% CI; both sexes combined)		1095·4 (1093·5–1097·3)	..	1151·1 (1149·2–1153·1)	..	1187·1 (1185·1–1189·1)	..	1224·4 (1222·3–1226·5)	..	1244·7 (1242·5–1246·9)	..	1262·6 (1260·3–1264·8)	..	1270·3 (1267·9–1272·6)	..	15·96% (15·69–16·23)	..	174·9 (172·0–177·7)	..

Data in parentheses are SEs, unless otherwise specified.

## References

[1] McCormick A, Fleming D, Charlton J (1995). Morbidity statistics from general practice: fourth national study 1991–1992.

[2] Hippisley-Cox J, Vinogradova Y (Sept, 2009). Trends in consultation rates in general practice 1995/1996 to 2008/2009: analysis of the QResearch® database. http://www.hscic.gov.uk/catalogue/PUB01077/tren-cons-rate-gene-prac-95-09-95-09-rep.pdf.

[3] Office for National Statistics (2009). General household survey 2007.

[4] Gibson J, Checkland K, Coleman A Eighth national GP worklife survey report. http://www.population-health.manchester.ac.uk/healtheconomics/research/Reports/EighthNationalGPWorklifeSurveyreport/EighthNationalGPWorklifeSurveyreport.pdf.

[5] Herrett E, Gallagher AM, Bhaskaran K (2015). Data resource profile: Clinical Practice Research Datalink (CPRD). Int J Epidemiol.

[6] Mathur R, Bhaskaran K, Chaturvedi N (2014). Completeness and usability of ethnicity data in UK-based primary care and hospital databases. J Public Health (Oxford).

[7] Williams T, van Staa T, Puri S, Eaton S (2012). Recent advances in the utility and use of the General Practice Research Database as an example of a UK primary care data resource. Ther Adv Drug Saf.

[8] Health & Social Care Information Centre (March, 2015). General and personal medical services, England, 2004–2014. http://www.hscic.gov.uk/catalogue/PUB16934.

[9] Health & Social Care Information Centre (2011). Rural/urban definition of GP practice: categorical. https://indicators.ic.nhs.uk/webview/.

[10] NHS England (Jan, 2016). GP patient survey. Survey results and other information. https://gp-patient.co.uk/surveys-and-reports.

[11] Health & Social Care Information Centre (Oct, 2014). Quality & Outcomes Framework (QOF) scores—2013–14. http://www.hscic.gov.uk/catalogue/PUB15751.

[12] Office for National Statistics (June, 2014). Population estimates for UK, England and Wales, Scotland and Northern Ireland, mid-2013. http://www.ons.gov.uk/ons/publications/re-reference-tables.html?edition=tcm%3A77-322718.

[13] National Cancer Institute (April, 2015). Statistical Methodology and Applications Branch, Surveillance Research Program. Joinpoint user's guide 4.2. http://surveillance.cancer.gov/joinpoint/Joinpoint_Help_4.2.0.0.pdf.

[14] Mukhtar T, Yeates DR, Goldacre MJ (2013). Breast cancer mortality trends in England and the assessment of the effectiveness of mammography screening: population-based study. J R Soc Med.

[15] Office for National Statistics (June, 2015). Population estimates for UK, England and Wales, Scotland and Northern Ireland. http://www.ons.gov.uk/ons/rel/pop-estimate/population-estimates-for-uk--england-and-wales--scotland-and-northern-ireland/index.html.

[16] Campbell JL, Fletcher E, Britten N (2014). Telephone triage for management of same-day consultation requests in general practice (the ESTEEM trial): a cluster-randomised controlled trial and cost-consequence analysis. Lancet.

[17] NHS England Improving general practice—a call to action. Evidence Pack. Aug, 2013–14. https://www.england.nhs.uk/wp-content/uploads/2013/09/igp-cta-evid.pdf.

[18] Holt TA, Fletcher E, Warren F (2016). Telephone triage systems in UK general practice: analysis of consultation duration during the index day in a pragmatic randomised controlled trial. Br J Gen Pract.

[19] Doran N, Fox F, Rodham K, Taylor G, Harris M (2016). Lost to the NHS: a mixed methods study of why GPs leave practice early in England. Br J Gen Pract.

