# Department of Error

**DOI:** 10.1016/S0140-6736(16)30691-2

**Published:** 2016-06-04

*Hobbs FDR, Bankhead C, Mukhtar T, et al, on behalf of the National Institute for Health Research School for Primary Care Research. Clinical workload in UK primary care: a retrospective analysis of 100 million consultations in England, 2007–14.* Lancet *2016; **387:** 2323–30*—This Article should have been published under a Creative Commons CC BY open access licence. This correction has been made as of June 2, 2016, and the printed Article is correct.

