# Department of Error

**DOI:** 10.1016/S0140-6736(16)30692-4

**Published:** 2016-06-04

*Thompson M, Walter F. Increases in general practice workload in England.* Lancet *2016; **387:** 2270–72*—This Comment should have been published under a Creative Commons CC BY open access licence. This correction has been made as of June 2, 2016.

