# Supplementary material for: Clinical workload in UK primary care: a retrospective analysis of 100 million consultations in England, 2007–14
Source: Lancet. 2016 Jun 4;387(10035):2323–30. doi: 10.1016/S0140-6736(16)00620-6 (PMC4899422; doi:10.1016/S0140-6736(16)00620-6)
Supplement: Supplementary appendix [file mmc1.pdf]

THE LANCET

Supplementary appendix

This appendix formed part of the original submission and has been peer reviewed. We post it as supplied by the authors.

Supplement to: Hobbs FDR, Bankhead C, Mukhtar T, et al, on behalf of the National Institute for Health Research School for Primary Care Research. Clinical workload in UK primary care: a retrospective analysis of 100 million consultations in England, 2007–14. *Lancet* 2016; published online April 5. http://dx.doi.org/10.1016/S0140-6736(16)00620-6.

Appendix 1: Denominator data: practices contributing data in each year

Year	2007/8	2008/9	2009/10	20010/11	2011/12	2012/13	2013/14
Number of practices reporting at beginning of year	362	364	362	357	342	326	308
Number of practices that begin reporting during year	5	4	2	5	4	4	8
Number of practices that stop reporting during year	3	6	7	20	20	22	45
Number of practices that start and stop reporting during year	0	0	0	1	0	0	2
Number of practices that report for entire year	359	358	355	338	322	304	265
Number of practices that report at any time during year	367	368	364	362	346	330	316
Mean number of patients in contributing practices in each year	9036.7	9120.6	9219.0	9313.4	9452.0	9604.8	9649.7
Mean person-years follow-up for contributing practices in each year	8358.2	8342.8	8463.0	8361.8	8490.9	8648.7	8201.1

Appendix 2: Denominator data: person-years of observation, by age, sex, and year

	2007/8		2008/09		2009/10		2010/11		2011/12		2012/13		2013/14		
	Male	Female	Male	Female	Male	Female	Male	Female	Male	Female	Male	Female	Male	Female	Total
0-4	83451	79445	85364	81209	86478	82417	86532	82440	85396	81544	83972	80259	75825	72504	1146837
5-14	181086	171986	180159	172143	179419	171474	175657	167725	170547	162783	166200	159034	151998	145679	2355889
15-24	181001	168902	182353	171722	183743	174945	180810	173518	174385	168534	169127	164415	153176	150872	2397504
25-44	444319	430931	439546	428252	436220	425855	422687	415212	403126	400720	390011	390787	350878	353884	5732428
45-64	407932	397593	411465	400914	413199	403364	408182	398910	391813	384869	377157	371578	340414	335821	5443210
65-74	125880	135352	128008	137498	131036	140224	129402	137897	128332	136909	129221	137989	119227	127613	1844589
75-84	76182	103786	76518	102402	77159	101501	75815	99275	73818	95715	72641	92476	66747	83635	1197671
85+	22572	50769	23362	51339	24068	51539	24350	50618	24243	49090	23821	47343	22094	42961	508169
Total	1522423	1538765	1526776	1545478	1531321	1551318	1503436	1525596	1451660	1480164	1412150	1443881	1280359	1312969	20626297

Appendix 3 Practice characteristics of contributing practices.

Variable	Total number of practices	Median	IQR	Number of practices	Proportion
**Practice population**					
**Total FTE GPs (excluding GP registrars)**	386.00	4.65	3.70 to 6.59		
**All Staff FTE**	346.00	12.00	8.51 to 17.20		
**Total Nurse FTE**	346.00	2.00	1.00 to 3.00		
**Advance Nurse FTE**	346.00	0.50	0.50 to 1.00		
**Extended Nurse FTE**	346.00	0.50	0.50 to 1.00		
**Practice Nurse FTE**	346.00	1.00	1.00 to 2.00		
**Direct Patient Care FTE**	346.00	0.80	0.40 to 1.75		
**Proportion with at least one GP Registrar (including a training practice)**	386.00			154.00	0.40
**Quintile of QOF achievement 2012/13**	385.00				
1				43.00	0.11
2				62.00	0.16
3				76.00	0.20
4				92.00	0.24
5				112.00	0.29
**Quintile of QOF achievement 2013/14**	385.00				
1				58.00	0.15
2				69.00	0.18
3				65.00	0.17
4				95.00	0.25
5				98.00	0.25

**Variable**	**Total number of practices**	**Median**	**IQR**	**Number of practices**	**Proportion**
**GP Patient Survey 2013/14 response rate quintile (GPPS)**	386.00				
1				34.00	0.09
2				63.00	0.16
3				90.00	0.23
4				125.00	0.32
5				74.00	0.19
**% White - English / Welsh / Scottish / Northern Irish / British quintile (from GPPS)**	386.00				
1				44.00	0.11
2				83.00	0.22
3				102.00	0.26
4				82.00	0.21
5				75.00	0.19
**Long-standing health condition % yes quintile (from GPPS)**	386.00				
1				79.00	0.20
2				80.00	0.21
3				79.00	0.20
4				90.00	0.23
5				58.00	0.15

Variable	Total number of practices	Median	IQR	Number of practices	Proportion
**Practice rurality definition**	390.00				
Hamlet and Isolated Dwelling - Less Sparse				1.00	0.003
Town and Fringe - Less Sparse				45.00	0.12
Town and Fringe - Sparse				7.00	0.02
Urban >10k - Less Sparse				325.00	0.83
Village - Less Sparse				11.00	0.03
Village - Sparse				1.00	0.003
**Region**	398.00				
North East				9.00	0.02
North West				65.00	0.16
Yorkshire & The Humber				17.00	0.04
East Midlands				13.00	0.03
West Midlands				41.00	0.10
East of England				41.00	0.10
South West				51.00	0.13
South Central				41.00	0.10
London				69.00	0.17
South East Coast				51.00	0.13
**Index of multiple deprivation (2010) from practice postcode**	398.00	16.34	10.37 to 25.20		

Appendix 4: Numbers of consultations (with a GP or nurse) by age, sex, and year*

	2007/08		2008/09		2009/10		2010/11		2011/12		2012/13		2013/14	
	Male	Female	Male	Female	Male	Female	Male	Female	Male	Female	Male	Female	Male	Female
0-4	466742	418819	488336	438596	514867	466557	503150	452325	488308	439110	496011	448179	455414	413142
5-14	335365	344067	348241	364451	356938	371837	349192	366608	333280	352347	346989	365041	313057	331075
15-24	323823	709950	341047	755765	349256	816199	339992	784984	331031	751818	331853	747168	302314	686778
25-44	1028570	2176158	1036744	2238379	1066885	2284398	1035322	2247583	1006744	2195454	1002871	2170830	894009	1963786
45-64	1632525	2241305	1670784	2286128	1719566	2334388	1705142	2348460	1667450	2302589	1649791	2256497	1478071	2042727
65-74	928827	1087349	946723	1108345	990074	1140335	972409	1120316	974254	1119208	990900	1131018	920190	1050718
75-84	748426	1047515	766369	1048932	789143	1060425	778304	1048381	773172	1037938	779958	1024446	727431	944709
85+	252877	551718	275394	574577	290384	591762	298878	603024	309161	608247	313686	611300	294066	564285
Total	5717155	8576881	5873638	8815173	6077113	9065901	5982389	8971681	5883400	8806711	5912059	8754479	5384552	7997220

* Crude numbers of consultations decline over time because there are fewer patients contributing data in the dataset

** Shaded rows highlight the age groups contributing to the largest number of consultations.

Appendix 5: Crude and adjusted (to the 2013 mid-year English population) GP consultation rates/10,000 person-years by age, sex, and year

	**2007/08**		**2008/09**		**2009/10**		**2010/11**		**2011/12**		**2012/13**		**2013/14**	
	**Male**	**Female**	**Male**	**Female**	**Male**	**Female**	**Male**	**Female**	**Male**	**Female**	**Male**	**Female**	**Male**	**Female**
**0-4**	36875.22	34166.29	38010.14	35296.64	39103.38	36446.54	39144.91	36242.58	39247.54	36484.90	41164.77	38429.41	40860.07	38126.91
**5-14**	14077.08	15260.55	14538.72	15906.15	15134.33	16371.27	15340.04	16761.59	15219.67	16715.33	16390.20	17854.94	15915.96	17397.91
**15-24**	13695.80	31284.81	14220.21	32354.07	14592.21	33355.78	14568.71	33395.65	14780.14	33606.85	15468.10	34277.04	15466.32	34382.72
**25-44**	18160.27	37198.79	18603.00	38402.62	19373.78	39739.91	19497.21	40262.78	19924.47	40703.16	20669.45	41356.42	20612.02	41758.20
**45-64**	28972.25	41233.22	29493.41	42029.73	30215.73	42606.30	30497.64	43511.62	31120.45	44287.10	32265.23	45337.03	32414.84	45938.80
**65-74**	48272.04	55317.39	48419.83	55616.26	49246.66	56060.18	49327.37	56549.51	50212.61	57101.95	51276.41	57654.72	51963.98	58704.19
**75-84**	64179.06	69622.97	65522.87	71033.80	66583.93	72311.52	67649.35	73722.66	69111.90	75936.57	71425.23	78197.09	73364.39	80787.03
**85+**	75499.04	78284.67	79314.60	80827.26	81590.13	82815.44	83972.72	87293.29	87460.67	91210.32	91483.87	95803.37	94730.43	99344.52
**Total**	26709.39	40119.75	27400.39	41105.37	28255.89	42044.64	28576.24	42740.16	29219.00	43426.83	30464.77	44525.80	30724.64	45093.98
**Adjusted consultation rate/10,000/Y**	**33545.08* (95% CI: 33524.47 to 33565.69)**		**34337.40 (95% CI: 34316.62 to 34358.19)**		**35177.52 (95% CI: 35156.55 to 35198.50)**		**35630.58 (95% CI: 35609.30 to 35651.85)**		**36211.83 95% CI: 36190.07 to 36233.59)**		**37322.90 95% CI: 37300.54 to 37345.25**		**37689.85* (95% CI: 37666.29 to 37713.40)**	

*An increase of 12.36%

Appendix 6: GP face-to-face surgery consultations, crude and adjusted (to 2013 mid-year English population) rates/10,000 person-years

	2007/08		2008/09		2009/10		2010/11		2011/12		2012/13		2013/14	
	Male	Female	Male	Female	Male	Female	Male	Female	Male	Female	Male	Female	Male	Female
0-4	34241.09	31681.19	35029.37	32513.46	35339.87	32837.57	35485.12	32813.80	35484.97	32950.01	36423.55	33910.39	35050.14	32656.31
5-14	13212.18	14305.77	13552.37	14827.28	13796.34	14903.06	14077.52	15405.39	13943.30	15304.56	14679.19	15985.85	13860.01	15119.27
15-24	12839.62	28984.66	13245.29	29743.33	13362.72	30184.79	13379.01	30250.97	13560.03	30375.93	13869.77	30229.73	13441.39	29269.39
25-44	16774.26	33804.60	17035.27	34568.92	17500.05	35197.77	17614.87	35680.76	17926.19	35918.40	18161.39	35685.37	17487.86	34896.75
45-64	26715.80	37531.29	26962.40	37906.50	27384.58	38023.86	27603.12	38812.51	28100.81	39379.33	28664.66	39606.92	28036.97	39248.06
65-74	43642.61	49244.86	43369.83	49077.77	43845.76	49145.08	43816.11	49469.46	44609.55	50021.33	44902.01	49725.41	44705.66	49696.82
75-84	54980.10	57254.47	55403.88	57784.95	56264.13	58535.95	57193.29	59732.60	58491.51	61984.37	59600.36	63061.74	60417.88	64273.17
85+	55990.19	53412.11	58535.81	54274.93	59811.60	55710.30	61890.84	59102.25	65276.55	62527.81	67687.78	65643.77	68989.08	67140.90
Total	24218.87	35536.18	24618.97	36075.33	25116.93	36467.78	25400.65	37086.63	25925.33	37629.18	26542.74	37882.33	26079.11	37409.73
Adjusted consultation rate/10,000PY	30028.68* (95% CI: 30009.17 to 30048.19)		30459.86 (95% CI: 30440.27 to 30479.45)		30850.35 (95% CI: 30830.69 to 30870.00)		31255.92 (95% CI: 31235.98 to 31275.85)		31718.72 (95% CI: 31698.34 to 31739.09)		32097.03 (95% CI: 32076.29 to 32117.77)		31589.53* (95% CI: 31567.95 to 31611.11)	

*An increase of 5.20%

Appendix **7**: GP telephone consultations: crude and adjusted (to the 2013 mid-year English population) rates/10,000 person-years

	2007/08		2008/09		2009/10		2010/11		2011/12		2012/13		2013/14	
	Male	Female	Male	Female	Male	Female	Male	Female	Male	Female	Male	Female	Male	Female
0-4	2523.40	2379.12	2875.81	2687.13	3663.14	3496.37	3587.45	3361.95	3704.84	3479.08	4684.30	4467.31	5764.43	5426.47
5-14	828.83	923.10	944.94	1042.74	1296.97	1430.30	1231.32	1326.10	1253.03	1385.53	1680.15	1839.60	2037.07	2256.12
15-24	812.59	2209.56	929.79	2526.24	1183.18	3080.10	1144.57	3068.04	1180.83	3162.21	1556.76	3982.42	1987.19	5060.64
25-44	1314.35	3236.02	1495.91	3678.66	1801.25	4403.01	1808.60	4455.58	1929.62	4668.72	2443.42	5557.86	3059.41	6757.36
45-64	1994.62	3327.50	2256.40	3740.16	2570.07	4227.80	2632.01	4350.41	2779.42	4572.75	3367.11	5409.40	4143.78	6383.67
65-74	3458.78	4635.54	3897.18	5124.14	4326.91	5642.49	4443.43	5799.09	4577.52	5883.19	5376.20	6754.07	6306.77	7883.20
75-84	5371.18	7059.31	6064.68	7887.24	6620.70	8643.19	6750.35	8928.35	7013.43	9095.48	8230.75	10353.65	9462.75	11862.83
85+	7733.10	9066.13	8770.12	10149.99	10067.00	11255.96	10303.78	11862.13	10460.75	12407.86	12087.58	13990.93	13946.55	15890.80
Total	1920.98	3417.57	2186.99	3843.29	2569.47	4448.65	2600.81	4525.35	2729.05	4700.88	3356.10	5568.08	4082.35	6627.38
Adjusted consultation rate/10,000PY	2668.80* (95% CI: 2663.00 to 2674.60)		3011.57 (95% CI: 3005.42 to 3017.71)		3504.89 (95% CI: 3498.28 to 3511.51)		3552.93 (95% CI: 3546.22 to 3559.64)		3696.53 (95% CI: 3689.59 to 3703.47)		4439.16 (95% CI: 4431.46 to 4446.87)		5327.77* (95% CI: 5318.92 to 5336.63)	

* 99.63% increase from 2007/2008

Appendix 8: Patient-level, univariate, zero truncated negative binomial regression analysis exploring consultation rates with IMD score in quintiles

	IRR	95% CI	% Change
IMD Score in quintiles			
1	0.8852	0.8812 to 0.8892	-11.5
2	0.9851	0.9806 to 0.9896	-1.5
4	0.9908	0.9861 to 0.9955	-0.9
5	1.0355	1.0303 to 1.0407	3.5

The above table shows that:

1. Patients in quintile 1 would be expected to have a consultation rate that was 11.5% lower those in quintile 3
2. Patients in quintile 2 would be expected to have a consultation rate 1.5% lower than those in quintile 3
3. Patients in quintile 4 would be expected to have a consultation rate 0.9% lower than those in quintile 3
4. Patients in quintile 5 would be expected to have a consultation rate 3.5% higher than those in quintile 3

Appendix 9: Duration of face-to-face GP consultations (minutes)

	2007/08		2008/09		2009/10		2010/11		2011/12		2012/13		2013/14		% change from 2007/08 – 2013/14		Change in seconds	
	Male	Female	Male	Female	Male	Female	Male	Female	Male	Female	Male	Female	Male	Female	Male	Female	Male	Female
0-4	7.91	7.83	8.21	8.09	8.35	8.24	8.62	8.49	8.67	8.58	8.75	8.65	8.86	8.78	11.99%	12.11%	56.88	56.93
5-14	7.29	7.45	7.55	7.69	7.72	7.87	7.94	8.09	8.00	8.13	7.95	8.19	8.13	8.32	11.61%	11.75%	50.78	52.53
15-24	7.96	8.61	8.22	8.85	8.36	9.05	8.63	9.31	8.72	9.36	8.66	9.33	8.77	9.49	10.10%	10.24%	48.26	52.88
25-44	8.81	9.09	9.09	9.34	9.19	9.48	9.40	9.66	9.45	9.66	9.34	9.59	9.43	9.68	6.99%	6.44%	36.97	35.15
45-64	9.04	9.10	9.30	9.33	9.39	9.45	9.56	9.62	9.53	9.60	9.45	9.54	9.48	9.63	4.80%	5.86%	26.07	32.00
65-74	8.54	8.62	8.87	8.90	8.97	9.03	9.15	9.18	9.07	9.11	9.01	9.04	9.08	9.17	6.26%	6.39%	32.10	33.03
75-84	8.44	8.49	8.71	8.77	8.76	8.88	8.96	9.05	8.89	8.99	8.82	8.90	8.91	9.00	5.62%	6.00%	28.44	30.55
85+	7.93	7.50	8.13	7.77	8.28	7.88	8.41	7.97	8.32	7.94	8.23	7.82	8.39	7.95	5.82%	6.08%	27.69	27.33
Total	8.65 95% CI (8.64, 8.65)		8.90 95% CI (8.90, 8.91)		9.03 95% CI (9.02, 9.03)		9.21 95% CI (9.21, 9.22)		9.20 95% CI (9.20, 9.20)		9.13 95% CI (9.13, 9.14)		9.22 95% CI (9.22, 9.23)		6.68%		34.64 secs	

Appendix 10: Total number of days (2 x 3 hour surgery sessions) per 10,000 person years spent doing face-to-face, telephone, or visit consultations with a GP

	2007/08		2008/09		2009/10		2010/11		2011/12		2012/13		2013/14		% change from 2007/08 – 2013/14		Change in days	
	Male	Female	Male	Female	Male	Female	Male	Female	Male	Female	Male	Female	Male	Female	Male	Female	Male	Female
0-4	784.5	720.5	836.0	766.1	869.1	799.7	895.7	817.2	901.8	828.8	942.1	869.4	932.0	861.6	18.8%	19.6%	147.5	141.1
5-14	278.2	307.8	297.0	330.4	314.1	345.5	326.8	363.7	326.0	363.6	345.7	386.6	338.5	377.8	21.6%	22.8%	60.2	70.0
15-24	295.2	723.0	315.5	765.8	327.0	802.5	336.9	825.4	345.1	833.5	354.7	837.7	353.8	840.0	19.9%	16.2%	58.7	117.0
25-44	430.3	902.1	452.8	952.0	473.9	994.2	487.5	1025.6	500.0	1035.2	507.8	1033.4	503.0	1038.5	16.9%	15.1%	72.7	136.3
45-64	706.0	1004.6	735.8	1045.5	758.9	1069.7	778.8	1110.6	793.1	1127.7	809.5	1139.4	807.1	1154.8	14.3%	15.0%	101.1	150.2
65-74	1109.9	1274.5	1150.5	1318.0	1182.6	1346.3	1206.5	1378.3	1220.5	1384.0	1231.3	1380.2	1248.4	1416.3	12.5%	11.1%	138.5	141.8
75-84	1442.7	1552.8	1513.3	1629.9	1547.7	1680.0	1608.9	1747.6	1638.0	1797.3	1674.2	1830.2	1731.3	1907.1	20.0%	22.8%	288.5	354.3
85+	1556.4	1514.4	1676.9	1612.5	1754.2	1681.8	1837.7	1799.9	1917.3	1893.8	1997.8	1979.0	2104.9	2087.6	35.2%	37.8%	548.5	573.2
Total	772.3 95% CI (771.0, 773.7)		812.0 95% CI (810.6, 813.4)		841.8 95% CI (840.4, 843.2)		870.8 95% CI (869.4, 872.3)		886.7 95% CI (885.2, 888.3)		902.6 95% CI (901.0, 904.2)		913.1 95% CI (911.4, 914.8)		18.2%		140.8	
